# Imaging of electrical activity in small diameter fibers of the murine peripheral nerve with virally-delivered GCaMP6f

**DOI:** 10.1038/s41598-018-21528-1

**Published:** 2018-02-19

**Authors:** Hans E. Anderson, Arjun K. Fontaine, John H. Caldwell, Richard F. Weir

**Affiliations:** 1 Department of Bioengineering, University of Colorado – Anschutz Medical Campus, Colorado, USA; 2 Department of Cell and Developmental Biology, University of Colorado – Anschutz Medical Campus, Colorado, USA

## Abstract

Current neural interfaces are hampered by lack of specificity and selectivity for neural interrogation. A method that might improve these interfaces is an optical peripheral nerve interface which communicates with individual axons via optogenetic reporters. To determine the feasibility of such an interface, we delivered the genetically encoded calcium indicator GCaMP6f to the mouse peripheral nerve by intramuscular injection of adenoassociated viral vector (AAV1) under the control of the CAG (chicken beta actin- cytomegalovirus hybrid promoter). Small diameter axons in the common peroneal nerve were transduced and demonstrated electrically inducible calcium transients *ex vivo*. Responses to single electrical stimuli were resolvable, and increasing the number of stimuli resulted in a monotonic increase in maximum fluorescence and a prolongation of calcium transient kinetics. This work demonstrates the viability of using a virally-delivered, genetically-encoded calcium indicator to read-out from peripheral nerve axons.

## Introduction

A robust interface with the nervous system in the periphery is a critical component in the restoration of function through limb reanimation in persons with spinal cord injury (SCI) and stroke^1,2^, control of prosthetic limbs for individuals with limb loss^3,4^, and in the exploration of peripheral neural circuits^5^.

Sensory feedback plays an important role in dexterous manipulation^6^, object identification^7^, pain^8^ the exercise pressor reflex^9,10^ and reduces the amount of visual attention required for manipulating objects with either prostheses or through limb reanimation (review^11^). As a variety of pathologies may disrupt sensory axons, neural interrogation of peripheral sensory axons is also useful in a clinical setting^12,13^.

Surface electroneurography, which is employed clinically in nerve conduction studies, only measures approximately the fastest 20% of sensory fibers, thereby ignoring a large number of both myelinated and unmyelinated fibers that may be affected in a disease state^14^. Surface electroneurograms also must contend with low signal strength, high noise, a limitation in the fiber size that can be read from, and distortion in signal shape^15^. While some of these factors can be mitigated by using penetrating, fine-wire electrodes to measure the sensory action potential, such a method is not a robust interface for long-term use in SCI patients. Moreover, in both clinical and research settings, it may not provide sufficient specificity for detailed read-out.

There are several more invasive techniques for read-out in the periphery. Nerve cuff electrodes offer some promise in stimulation ability and serve as a backbone for functional electrical stimulation^3,16^. However, they do not provide an effective means of interrogating small fibers, as signal recording becomes dominated by large fibers. Their ability to interrogate individual fibers or fibers at the subfascicular level is also limited^4^. Penetrating electrodes such as the Utah Slant Array^17^, the Michigan Array^18^ and the longitudinal intrafascicular electrode, have limited numbers of channels, can cause nerve damage, or suffer from biocompatibility issues that reduce their long-term viability^4,19^.

The use of optical techniques may provide an improved method of noninvasively sending and receiving signals from the nervous system through light-sensitive protein actuators such as channelrhodopsin-2^5,20^, and reporting of changes in cell parameters such as calcium^21,22^, voltage^23,24^ or pH^25,26^. These actuators and reporters have been used extensively in cultured neurons, zebrafish, and mice, and could form the basis of an optically based neural interface that avoids physically damaging nerve tissue^27^. Numerous individual axons may be labeled and thereby provide axon-level resolution, a substantial benefit over electrode systems that may be limited to a handful of electrodes.

Calcium transients, which can be used as an indicator of action potentials (APs), have been demonstrated in response to activity in somas^27–29^ and axons of peripheral nerves, including in C fibers^30^ and motor axons^31,32^. Thus, calcium could be used as an indicator of neural activity. Because calcium sensors tend to be substantially brighter^33^ and provide signal from entire sections of axoplasm, they may be more robust than membrane bound voltage sensors for imaging signal in the highly scattering environment of the densely myelinated peripheral nerve.

With the growing use of adenoassociated viral vectors (AAVs) in clinical trials (review^34,35^), and their demonstrated use for targeting various tissues through selection of serotype, promoter, and route of administration, it is now possible to selectively express an optical reporter protein in an adult wild-type animal. One particularly useful route of administration for a peripheral nerve interface is intramuscular injection, which can be done relatively noninvasively; retrograde transport from the muscle to the innervating fibers will confer expression to a select number of axons^36–38^. The benefit of this method over other methods is the restriction of expression largely to nerves innervating the injected muscle^5^, decreasing concerns of off-target expression in other tissues that can occur with intravenous injections^39^, and avoiding the surgical complications of nerve and spinal cord injections. These fibers potentially could be labeled with spectrally-separated reporter proteins to provide for additional discrimination between fibers innervating different muscles during interrogation.

Towards this end, we have injected mice intramuscularly with an AAV1 construct carrying a calcium sensor with rapid kinetics, GCaMP6f^22^, under the control of the chicken beta actin-cytomegalovirus hybrid (CAG) promoter. We excised peripheral nerves and performed electrical stimulation with concurrent fluorescence imaging using a variety of stimulation parameters relevant to physiological parameters.

## Results

### Intramuscular injection of AAV1-CAG-GCaMP6f results in GCaMP6f expression in small diameter axons in the common peroneal nerve

Five mice were injected bilaterally in the anterior tibialis muscle. A cursory search of the approximately 30 μm of optical depth reachable by the microscope in the common peroneal nerve (diameter ~300 μm) discovered 79 axons with 9 of 10 nerves showing GCaMP6f expression in at least one axon (average number of axons expressing GCaMP6f per nerve was 8.78 ± 1.73 S.E.M.). Not all of these axons were responsive to stimulation, and were thus excluded from further study (see below and Supplementary Table S1). All injections into the muscle were confirmed following excision of the tibialis anterior and imaging of co-injected fluorescent polystyrene beads. In all injections, expression was seen in the muscle tissue (Supplementary Fig. 1). Peroneal axons expressing GCaMP6f were generally small in diameter, with a mean diameter of 1.08 ± 0.36 μm (S.D.). Almost half (48%) of these axons were less than 1μm in diameter (Supplementary Fig. 2). Their small diameters suggest that they are either type III (thinly myelinated) or IV (unmyelinated) afferents (review^8^).

### Calcium influx occurs in small diameter axons following electrical stimulation

**Figure 1:**
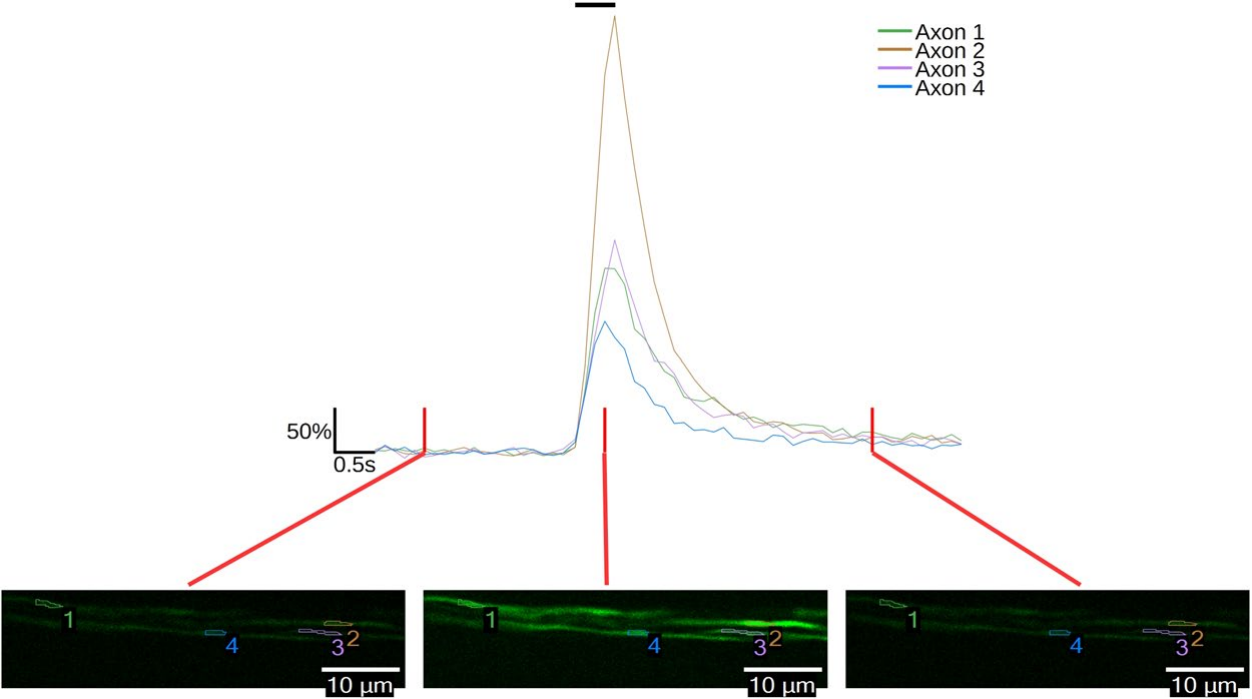
GCaMP6f reports neural activity in small diameter axons of the mouse common peroneal nerve. Traces of GCaMP6f fluorescence following a 50 Hz, 500 ms electrical stimuli train for four regions of interest from four axons identified in false color fluorescence images at the bottom, showing fluorescence before the stimulus train (left), immediately at the end of the stimulus train (center), and 3.75 seconds after the stimulus train (right). Time points corresponding to each image are indicated with a vertical red line. A black bar at the top of the traces indicates the time of stimulus. Fluorescence change is reported as percent ΔF/F_0_.

Electrical stimulation of either the sciatic nerve or common peroneal nerve via glass suction electrodes with 30 μs pulses at 50 Hz produces a transient calcium elevation in small diameter axons of the common peroneal nerve, as reported by GCaMP6f (Fig. 1 and Supplementary Video 1). These transients appeared approximately uniformly throughout the length of the axon, never appearing to originate at a single point.

### Small numbers of electrical stimuli can be resolved in small diameter axons of the peripheral nerve using GCaMP6f

**Figure 2:**
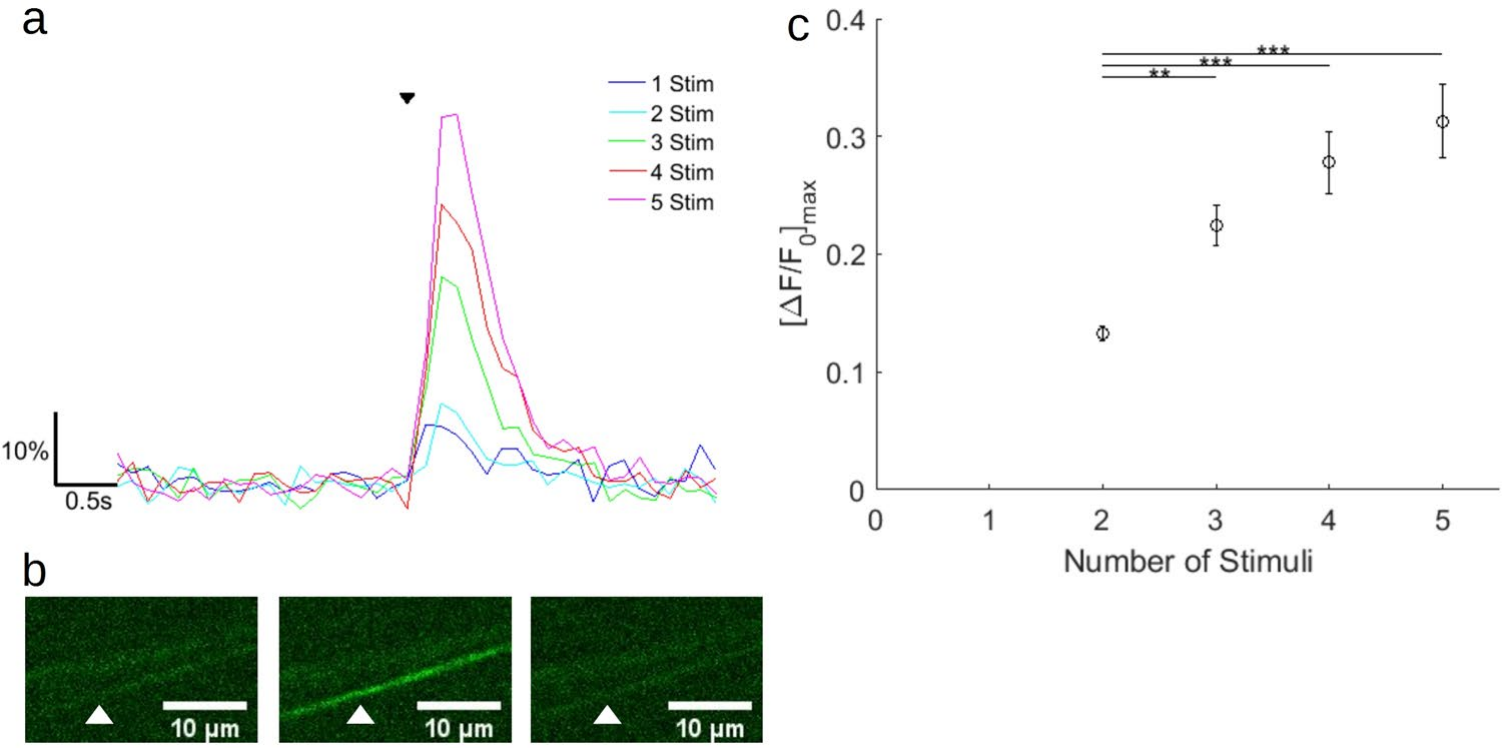
GCaMP6f detects small numbers of electrical stimuli in small diameter axons of the mouse common peroneal nerve. (**a**) Representative fluorescence traces from averages of six trials from two axons. Stimulus at 100 Hz for 1–5 stimuli indicated by small black arrowhead. Fluorescence is reported as percent ΔF/F_0_. 1–2 stimuli were from one axon, 3–5 were from another. (**b**) Images from a single trial of 5 stimuli. Left, before stimulus. Center, at end of stimulus train. Right, 2.5 s after stimulus train. White arrowhead indicates axon responding to 5 stimuli displayed in (**a**). (**c**) The mean peak response of axons to small numbers of stimuli (n = 7 axons from 5 mice). Error bars represent mean ± S.E.M. (Kruskal-Wallis nonparametric analysis of variance, p = 7.88e-07). Bars indicate significant results from Tukey-Kramer multiple comparison. **P < 0.01, ***P < 0.001. (P-values presented in Supplementary Table S2).

Two to five stimuli cause a calcium transient in the small fibers of the peripheral nerve. In two axons, a response to a single stimulus was detected, with an increase in signal of ~10% over baseline (Fig. 2a). Response of an axon to a five 30 μs stimulus train is shown in the images of Fig. 2b. As the number of stimuli increases (30 μs stimulus duration at 100 Hz), a roughly linear relationship between the number of stimuli and the maximum fluorescence change ensues (Fig. 2c; Kruskal-Wallis nonparametric analysis of variance, p = 7.88e-07; Supplementary Table S2).

### Increasing the number of electrical stimuli in a train increases signal and delays kinetics

**Figure 3:**
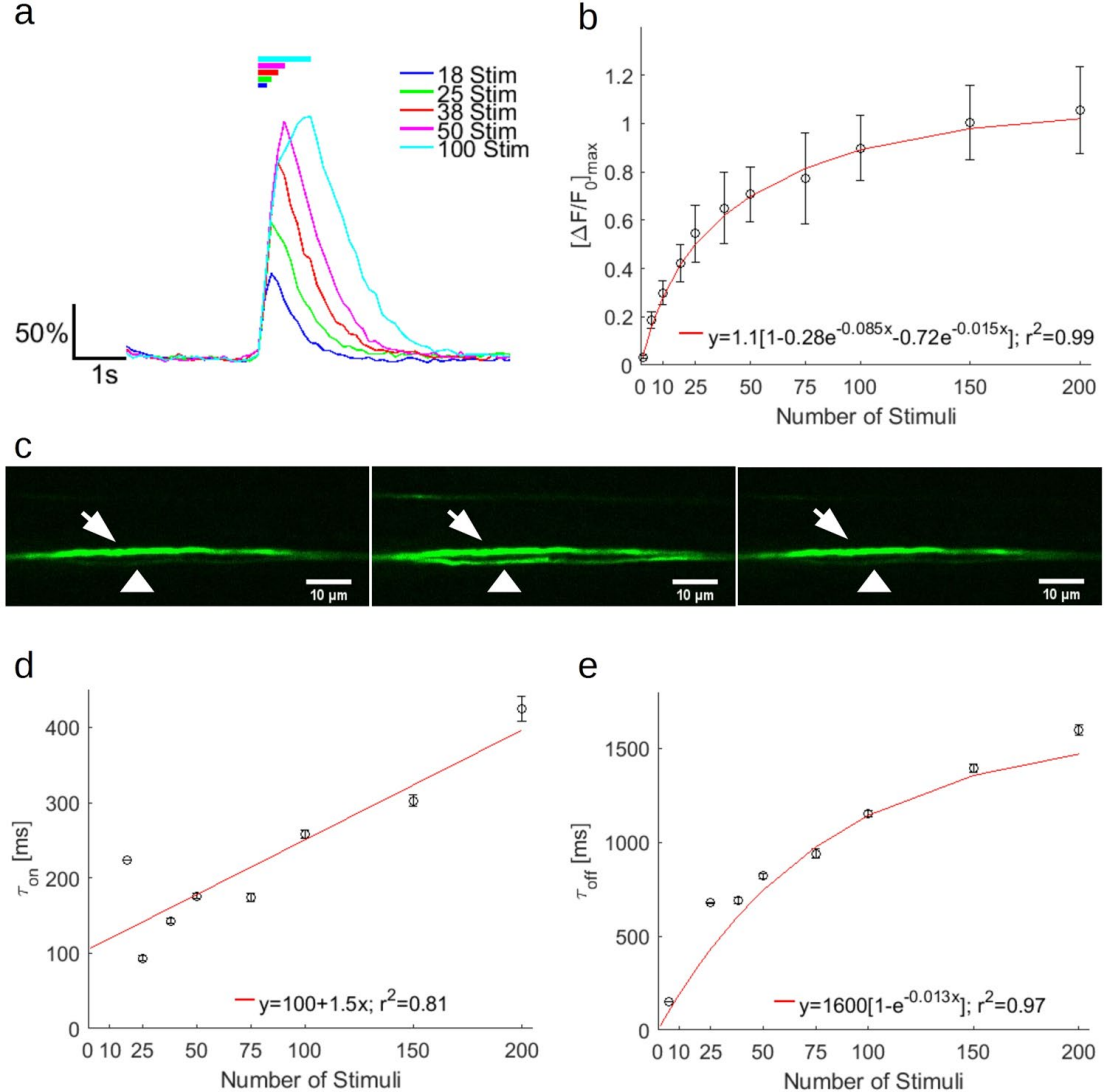
Increasing the number of stimuli at constant frequency increases maximum fluorescence change, and slows kinetics of GCaMP6f in small diameter axons. (**a**) Representative fluorescence traces from averages of six trials from a single axon, for varying numbers of stimuli at a constant frequency of 100 Hz. Fluorescence is reported as percent ΔF/F_0_. Durations of the stimulus train are indicated as solid, color coded bars at the top of the image. (**b**) Mean peak fluorescence of following 1–200 stimulus trains. A double exponential fit is indicated with a red line. (Kruskal-Wallis nonparametric analysis of variance, p = 3.71e-21). (**c**) Images from a single trial of 100 stimuli. Left, before stimulus. Center, at end of stimulus train. Right, 3.75 s after stimulus train. White arrowhead indicates axon in (**a**). White arrow indicates axon unresponsive to stimulus. (**d**) Rise time constant in ms computed from single exponential fits of averages from each axonal response to 1–200 stimuli. A linear fit is indicated with a red line. (Kruskal-Wallis nonparametric analysis of variance, p = 1.13e-12). (**e**) Off time constant in ms computed from single exponential fits of averages from each axonal response to 1–200 stimuli. (Kruskal-Wallis nonparametric analysis of variance, p = 2.69e-11). (**b**,**d**,**e**) Error bars represent mean ± S.E.M. (n = 22 axons from 4 mice). (P-values presented in Supplementary Table S3).

Increasing the number of 30 μs stimuli (by increasing the duration of a 100 Hz train) results in an increase in the maximum fluorescence. In traces from a single axon showing some of the largest [ΔF/F_0_] (selected for clarity, other axons showed similar shapes), general features can be observed (average of six trials, Fig. 3a; single trial shown in Fig. 3c). For example, even with a train duration of 1000 ms (100 stimuli), no plateau is reached. Furthermore, as the number of stimuli increases, the fluorescence peak continues to shift further to the right, suggesting either continual, increasing intraaxonal calcium concentrations, or summation effects due to GCaMP6f kinetics (see discussion). The peak fluorescence rapidly increases until 50 stimuli, where the rate of increase begins to slow, reaching a [ΔF/F_0_]_max_ = 0.876 ± 0.113 at 200 stimuli (Fig. 3b; Kruskal-Wallis nonparametric analysis of variance, p = 3.71e-21; Supplementary Table S3). This increase can be fitted with a double exponential (red curve in Fig. 3b). Similarly, the ‘on’ time constant, computed by curve fitting a single exponential function to the rise, shows a trend of increasing ‘on’ time constants with increasing numbers of stimuli (Fig. 3d; Kruskal-Wallis nonparametric analysis of variance, p = 1.13e-12; Supplementary Table S3); this is reflected in the rightward shift of the fluorescence peak towards the end of the stimulus train. This trend can be fitted with a linear regression (red line in Fig. 3d). It should be noted that the predictive value of this trend is limited at time constants shorter than the imaging frequency (~125 ms). The ‘off’ time constant, obtained by fitting the decay from the peak of the averaged trace for each axon, also shows a prolongation with increasing numbers of stimuli (Fig. 3e; Kruskal-Wallis nonparametric analysis of variance, p = 2.69e-11; Supplementary Table S3), exhibiting an asymptotic behavior that can be fitted with a single exponential (red line in Fig. 3e). Plotting the off-time constant versus axon prestimulus brightness does not show a statistically significant correlation across stimuli groups between axon brightness and off-time constant (Supplementary Fig. S3; Supplementary Table S4).

### Increasing the frequency of a stimulus train increases calcium influx and delays kinetics

**Figure 4:**
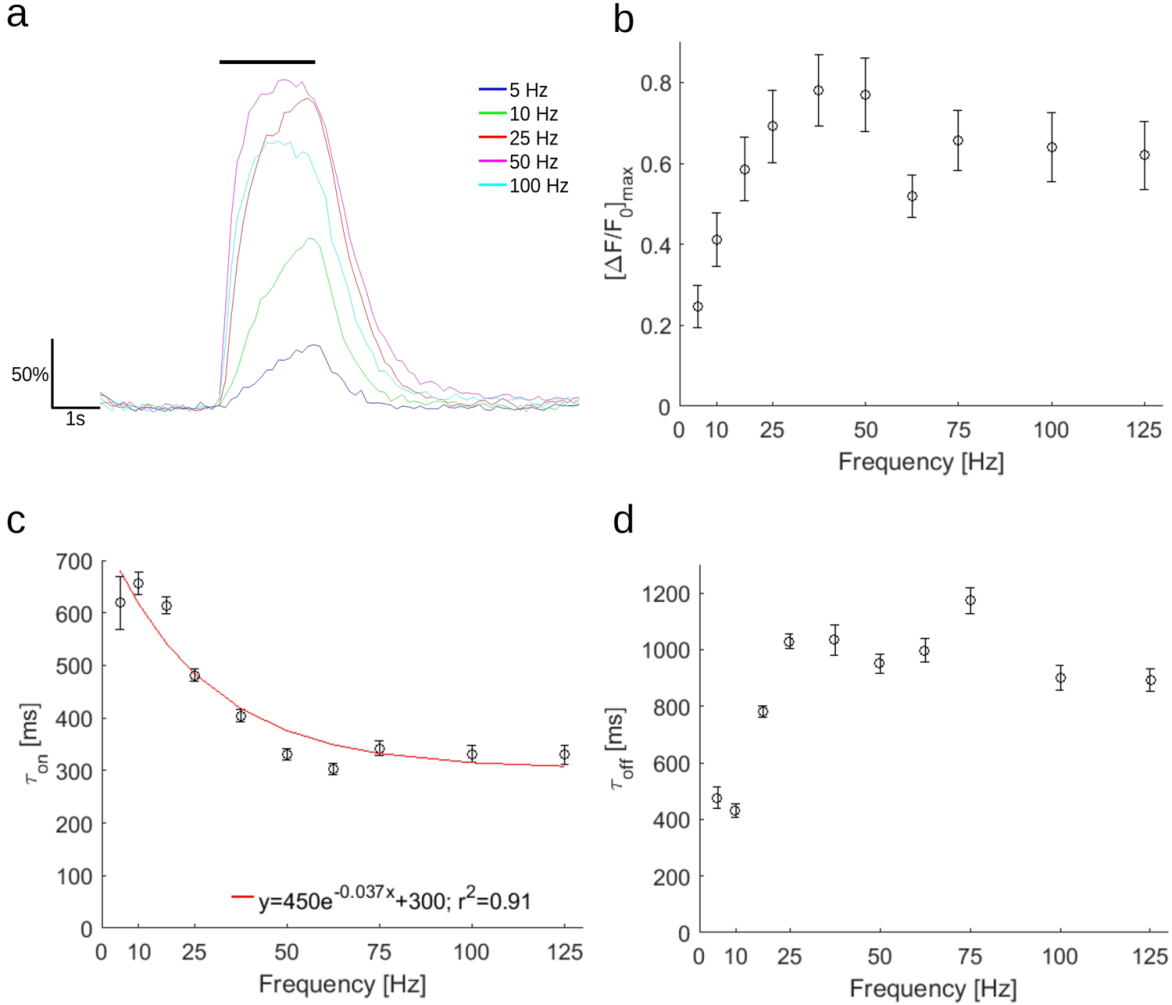
Increasing the frequency of a constant duration stimulus has a nonlinear effect on peak fluorescence change and kinetics of GCaMP6f in small diameter axons. (**a**) Representative traces from averages of six trials from a single axon for varying frequencies of electrical stimulus trains with a train duration of 2 s. A black bar at the top of the image indicates stimulus train duration. Fluorescence is reported as percent ΔF/F_0_. (**b**) Mean peak fluorescence following stimulation with 5–125 Hz. (Kruskal-Wallis nonparametric analysis of variance, p = 0.1246). (**c**) Rise time constant in ms computed from single exponential fits of averages from each axonal response to 5–125 Hz. A single exponential fit is indicated with a red line. (Kruskal-Wallis nonparametric analysis of variance, p = 1.13e-05). (**d**) Off time constant in ms computed from single exponential fits of averages from each axonal response to 5–125 Hz. (Kruskal-Wallis nonparametric analysis of variance, p = 4.89e-06). (**b**,**c**,**d**) Error bars represent mean ± S.E.M. (n = 14 axons from 3 mice). (P-values presented in Supplementary Table S5).

In fluorescence traces from a single axon stimulated with 30 μs stimulus trains of 2 s of varying frequency from 5 Hz to 125 Hz, increasing frequency first raises the peak fluorescence, which occurs near the end of the stimulus train, but as the frequency continues to increase, the curve flattens during the stimulus period, and the peak fluorescence moves closer to the initiation of the stimulus. Furthermore, above 50 Hz, the peak decreases (Fig. 4a). In aggregate, however, no statistical difference between frequencies across all axons for the various frequencies could be detected (Kruskal-Wallis nonparametric analysis of variance, P = 0.125), although a general plateau shape is presented (Fig. 4b). The ‘on’ time constant versus frequency follows an exponential decay (fit: y = 450e^−0.037x^ + 300, r^2^ = 0.91), reaching an asymptote at high frequencies (Fig. 4c; Kruskal-Wallis nonparametric analysis of variance, p = 0.127; Supplementary Table S5). The decay time constant, increases with frequency until about 37.5 Hz, after which it reaches a plateau (Fig. 4d; Kruskal-Wallis nonparametric analysis of variance, p = 1.13e-05; Supplementary Table S5). Plotting the initial slope versus the frequency yields a similar shape to Fig. 4b (Supplementary Fig. S4; Kruskal-Wallis nonparametric analysis of variance, p = 0.41).

## Discussion

We have demonstrated expression of GCaMP6f in small diameter axons of the murine peripheral nerve and measured robust fluorescence transients in response to electrical stimulus trains in the *ex vivo* preparation. We believe this is the first report of electrically-induced calcium transients visualized with a virally-delivered fluorescent protein in the peripheral nerve. We have expressed our results in terms of stimulus number rather than number of action potentials. Because these stimuli trains are in a frequency range that unmyelinated (C-fibers) and thinly myelinated (Aδ) fibers are known to follow^40,41^, we believe the number of stimuli is closely related to the number of action potentials at least up to 100 Hz in unmyelinated and thinly myelinated fibers. Signals from small numbers of electrical stimuli can be resolved, and the increase due to each additional stimulus appears to be linear (Fig. 2c). This linear trend, however, begins to plateau at longer duration stimulation, suggesting either the approach to a balance between calcium influx and efflux at these longer durations, a maximum physiological calcium influx, or saturation of the sensor. We were unable to calibrate the concentration of a non-ratiometric sensor like GCaMP in the axon, which may have varying levels of expression. Given the barriers of epineurium, perineurium, and ensheathing Schwann cells, it is difficult to apply bath solutions of detergents to attempt to permeabilize the membrane and obtain a single axon’s maximum fluorescence; this would have allowed us to conclusively determine if a higher fluorescence could be achieved under nonphysiologic conditions^30,42^ and thereby exclude sensor saturation as the cause of the plateau. A similar nonlinear response can be seen with increased numbers of action potentials in murine postganglionic sympathetic axons using dextran-conjugated Oregon Green 488 BAPTA-1^43^ and in unmyelinated axons of the rat vagus nerve^42^, suggesting a potentially similar mechanism. This plateau may be caused by regulation of action potential frequency to prevent damage^30,42–44^. Regardless of the underlying mechanism, the maximum GCaMP6f fluorescence would not preclude the utility of the indicator in reporting activity, provided that the pertinent range of frequency and duration did not exceed the dynamic range, or that a plateau was acceptable.

Generally, the rise and fall times of calcium transients are determined by the routes of influx and efflux, as well as, sequestration and buffering processes and temperature, concentration, and membrane conductances. In small, unmyelinated fibers this means N- and T-type calcium channels for influx^45,46^, and possibly ATP driven calcium pumps for efflux^43^, with endogenous buffering, and little role of calcium-induced calcium release. Additionally, GCaMP6f’s kinetics may be modifying the nature of the reported calcium signal, substantially integrating or summing the underlying calcium dynamic^22,27,47,48^. GCaMP6f has been shown to have slowed off-time kinetics (t_1/2_ ~ 288 ms, which would correspond to τ_off_ ~ 415 ms) and an increased dissociation constant at 20 °C^48^. Given that the experiments in this study were undertaken at 23 °C, the temperature alone could explain the off-time constants seen at low numbers of stimuli or at low frequencies, and the exaggeration with respect to the shorter processes seen in dendrites^49^. However, at higher numbers of stimuli, which increases the magnitude of the fluorescence change, and thusly the calcium transient, there is a prolongation of the off-time constant, above that suggested directly by the temperature. Since a linear relationship has been reported between binding capacity and buffer concentration, and therefore off-time constant^50,51^, increasing buffer concentration (which is proportional to fluorescence^52^) could result in delayed off kinetics, but it does not seem to be significant here (see Supplementary Fig. 3, Supplementary Table S4). Other endogenous processes involving reestablishment of intraaxonal calcium ion concentration which slow during long stimulation trains, such as those in cultured dorsal root ganglion cells, may be playing a role^44^. Similar endogenous effects causing prolongations of ‘off’ time constants can be seen from nociceptive fibers of human sural nerves using ratiometric dyes^30^, murine postganglionic sympathetic axon bundles using a nonratiometric dye^43^, and unmyelinated fibers of the rat vagus nerve^42^.

Integrative or summative effects may also be affecting the on-time constants. Such an effect may be demonstrated by the difference in on time constants between the duration and frequency trials. Chen *et al*.^22^ suggest that if the individual spikes of a stimulus train are separated by 75 ms (the ‘on’ time constant of GCaMP6f), they should be resolvable. Such a feature may be apparent in the averaged traces for low frequency stimuli (5–10 Hz, corresponding to 200–100 ms between pulses), and the appearance of a rightward shifted peak, following an incremental increase over the stimulus duration. As the frequency is increased, the peak shifts leftward, as the sensor fails to distinguish between pulses, instead aggregating the calcium signal. Since, even in short trains, a 100 Hz stimulus train greatly exceeds the on-rate of GCaMP6f, no such decrease in on-time constant is seen, being replaced by a linear increase in on-time suggesting a summation effect, as the fluorescence peak moves rightward. In addition, since GCaMP variants have been shown to have on time constants that are concentration dependent^53,54^, and the camera frame rate effectively bins calcium transients when the stimulus train frequency exceeds that of the camera, apparent on-time constants may be exaggerated in such cases, which may distort the suggested linear calcium accumulation from individual action potentials^49^.

In contrast to large myelinated fibers of the sciatic nerve^32^, but similar to the small myelinated fibers of the optic nerve^55^, the calcium transient in small fibers appears to occur along the entire length of the fiber. This difference is likely due to the distribution or accessibility of calcium channels – in the myelinated fibers they may be more common at the node^56^, or be blocked by the myelin^31^. A previous immunohistochemical study demonstrates T-type calcium channels along the length of the axon^45^, which could be responsible for the spatial extent of the calcium transient.

Interestingly, the vector appeared to transduce small diameter fibers of the peripheral nerve, at the exclusion of larger fibers. These fibers are likely either Group III or IV afferents; based on their size, many are likely to be unmyelinated and function as nociceptors, metaboreceptors, and thermoreceptors^8,27,57–59^. Given that AAV serotype 1 has been used successfully to target large diameter axons of the peripheral nerve using the cytomegalovirus promoter with intramuscular injections^36^, this transduction profile was likely the consequence of using the CAG promoter. A previous report^60^ showed expression in the somas of spinal cord motorneurons following intranerve injection of an AAV vector using the CAG promoter. It is then possible that the CAG promoter acts like its chicken beta actin (CBA) promoter parent, which only expresses at a low level in motorneurons^61^; in the earlier report, the concentration was sufficient to demonstrate some expression in the soma, but not in the axons, which may have occurred here. Only about 19% of the entire nerve was searched for expression, yielding 8.78 labeled axons. It is likely that more axons of the ~304 unmyelinated ones innervating the tibialis anterior^62^ lie too deep to be imaged with this setup and would require greater shaping of the nerve (such as through a cuff) or a technique such as two photon^63^, or three photon^64^ imaging and the application of a spatial light modulator^65^. A future study could better examine the effect of CAG promoter as well as the transduction efficiency of AAV1-CAG vectors in the fibers of the peripheral nerve.

Being able to read-out from these small fibers would greatly enhance the capabilities of neural interfaces for neural prostheses, such as those being developed for limb reanimation in the treatment of spinal cord injury. Current devices lack sensory feedback (review^2^), which is visually demanding for the patient in situations that make intuitive control difficult. Loss of nociception can lead to significant health risks^66^, and given the critical roles that thermoreceptors and metaboreceptors play in exercise physiology^9,10^, they will be imperative for restoration of unsupervised function in the natural environment. Optically-based techniques for limb animation and control of muscles have already been demonstrated (review^67^). Given the advantages of these methods over electrodes in terms of nerve damage, decreased muscle fatigue, and specificity, a natural extension of an optical interface for read-in is to combine with appropriate sensory information. This development will be a critical step in restoring function in the environment. An optical read-out system could provide an ability to read-out from single fibers, as demonstrated here, giving improved specificity without invasive and potentially damaging electrodes.

It may also be possible to employ optical interrogation for functional measurements in a research setting. Since calcium influx occurs during nerve injury, in both myelinated and unmyelinated axons (the potential cause of high brightness of the unresponsive axon in Fig. 3c; review^68^), it may be possible to use an optical nerve interface to report damage states, and generate a more continuous understanding of nerve damage than what might be possible with a measure such as the sciatic functional index. Furthermore, because it is possible to transduce motor and sensory axons selectively, independent measures of motor and sensory damage could be reported independently, and lesion affects more specifically to different anatomical regions.

This work demonstrates the viability of using AAVs to modify nerves in adult animals such that they express optical reporter proteins, enabling optical methods to be retroactively used for neural read-out.

## Methods

### Materials

Adenoassociated viral vector (AAV1.CAG.GCaMP6f.WPRE.SV40) was obtained from the University of Pennsylvania Vector Core. Fluorescent beads (2 μm, polystyrene, excitation 575 nm, emission 610 nm) were obtained from Sigma Aldrich; approximately 4 × 10^5^ beads were injected at each site.

### Intramuscular Injections

All animal procedures have been approved by the University of Colorado Anschutz Medical Campus Institutional Animal Care and Use Committee (IACUC) with accreditation by Association for Assessment and Accreditation of Laboratory Animal Care (AAALAC). All experiments were performed in accordance with the approved protocol and relevant IACUC regulations and guidelines.

Eleven-week-old, female C57BL/6 J mice (Jackson Laboratories) were initially anesthetized in a chamber with oxygen flow with 4–5% isoflurane. Once mice are anesthetized, as confirmed by toe pinch, they are transferred to a heating pad at 37 °C, and to a nose cone delivering 1.5–2% isoflurane. Skin overlaying the tibialis anterior is shaved using an electric razor and wiped with a betadine wipe. Mice are then injected in each anterior tibialis with 10^11^ viral genomes (vg) of viral vector and fluorescent beads. Following injections, mice are again wiped and allowed to recover in their cage. The following day, ambulation was checked.

### Electrophysiology

After five weeks, mice are sacrificed using isoflurane followed by cervical dislocation. Sciatic and attached peroneal nerves and anterior tibialis muscles were removed. Muscles were placed in 1× Phosphate Buffered Saline. Nerves were placed in a saline solution (in mM: NaCl 126, KCl 3, CaCl_2_ 2, MgCl_2_ 2, MOPS 10, Glucose 30; pH 7.25) in a glass bottomed imaging chamber equipped with two glass electrodes, one fitted to the sciatic nerve, the other fitted to the common peroneal nerve. The nerve was held in place in the imaging chamber using a U-shaped bent steel wire with nylon threads strung across the open section of the U. Electrical stimulation was provided using stimulus from either the sciatic or common peroneal electrodes using a Grass SD9 stimulator. Prior to conducting experiments, the electrodes and setup were evaluated for ability to generate compound action potentials (MultiClamp 700B Amplifier, Axon Instruments, PCLAMP10 Software). Compound action potentials were only measured as a part of hardware design, as the compound action potential does not report the activity of a single axon, only the aggregate of the nerve, reducing its utility for correlating single axon activity with calcium transients. Stimulus duration for eliciting an action potential was 30 μs. Stimulus frequencies ranged from 5–125 Hz based on previous reports of observed frequencies in type III (thinly myelinated) and type IV (unmyelinated) fibers following either chemical, mechanical, or thermal stimulation (1–200 Hz)^10,69–71^ and included most of the stimulus range attempted on other small fibers in other studies of the peripheral nerve (1–50 Hz)^30,42^. Stimulation voltages were the minimum required to elicit a change in GCaMP fluorescence and ranged from 5.5–15 V.

### Imaging

Imaging was performed using an inverted spinning disk microscope (Intelligent Imaging Innovations) equipped with a 488 nm laser and a 16-bit EMCCD camera. Frames were captured using a 63X oil objective (1.4NA) at ~8 Hz. Imaging experiments were performed at 23 °C.

### Data Analysis

Images were analyzed using SlideBook (Intelligent Imaging Innovations). Curve fitting, plotting and statistical analysis were completed using MATLAB (Mathworks). Generally, a Kruskal-Wallis Nonparametric Analysis of Variance was used in comparisons of more than two means, unless an Anderson-Darling test demonstrated normality, in which case a one-way analysis of variance was used. Multiple comparisons, conducted if analysis of variance tests showed significance, used the Tukey-Kramer method. P-values less than 0.05 were considered significant. Fluorescence changes were computed as ΔF/F_0_ = (F − F_intial_)/F_initial_, where *F* is the fluorescence at a point in time, *F*_*initial*_ is the mean fluorescence for 15 time points before stimulation. Rise and decay time constants were computed from averaged traces from individual axons fitted with single exponential curves. A Spearman’s correlation was used to compute the correlation between on-time constant and prestimulus axon brightness. To control family-wide error rate, the Benjamini-Hochberg method for controlling the false detection rate was used.

### Data Availability

The data generated during the current study are available from the corresponding author on reasonable request.

## Electronic supplementary material

Supplementary Video 1

Supplementary Information
