# Supplementary material for: Imaging of electrical activity in small diameter fibers of the murine peripheral nerve with virally-delivered GCaMP6f
Source: Sci Rep. 2018 Feb 19;8:3219. doi: 10.1038/s41598-018-21528-1 (PMC5818512; doi:10.1038/s41598-018-21528-1)
Supplement: Supplementary file 2 — Supplementary Information [file 41598_2018_21528_MOESM2_ESM.pdf]

Imaging of electrical activity in small diameter fibers of the murine peripheral nerve with virally-delivered GCaMP6f

Hans E. Anderson,[1] Arjun K. Fontaine,[1] John H. Caldwell,[2] Richard F. Weir[1]

[1]Department of Bioengineering, University of Colorado – Anschutz Medical Campus, Colorado, USA.
[2]Department of Cell and Developmental Biology, University of Colorado – Anschutz Medical Campus, Colorado, USA.

Supplementary Information

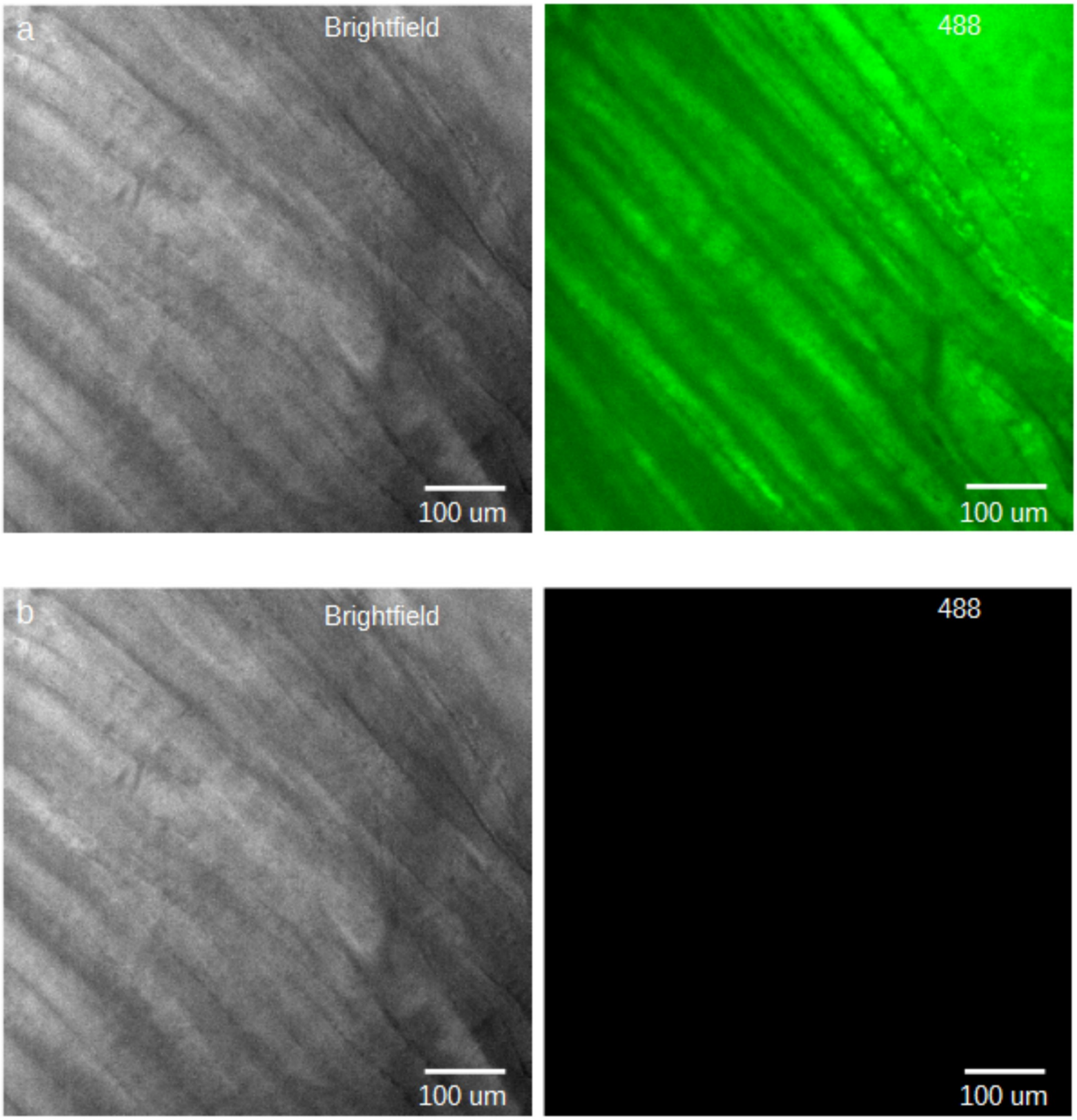

Supplementary Figure S1. Intramuscular injection of AAV1-CAG-GCaMP6f causes expression of GCaMP6f in injected muscle. (a) Brightfield (left panel) and spinning disk image (488nm excitation, right panel) of tibialis anterior muscle, injected with AAV1-CAG-GCaMP6f. (b) Brightfield and spinning disk image (488 excitation) of tibialis anterior muscle sham-injected with saline.

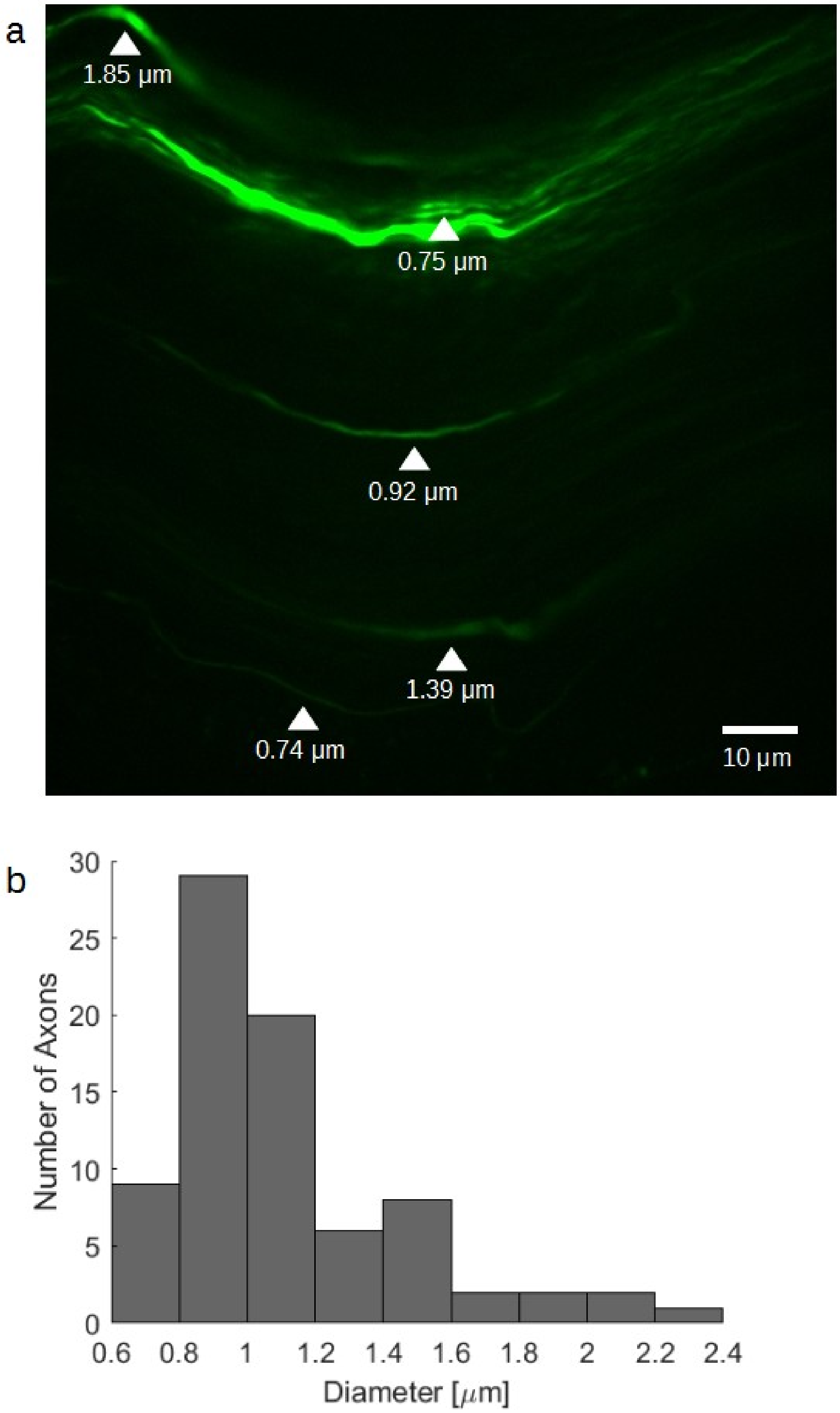

Supplementary Figure S2. <u>Expression profiles in the common peroneal nerve of GCaMP6f labeled axons.</u> (a) Five labeled axons with diameter measurements. (b) Histogram of diameters of GCaMP6f-expressing axons (n=79 axons from 5 mice).

a

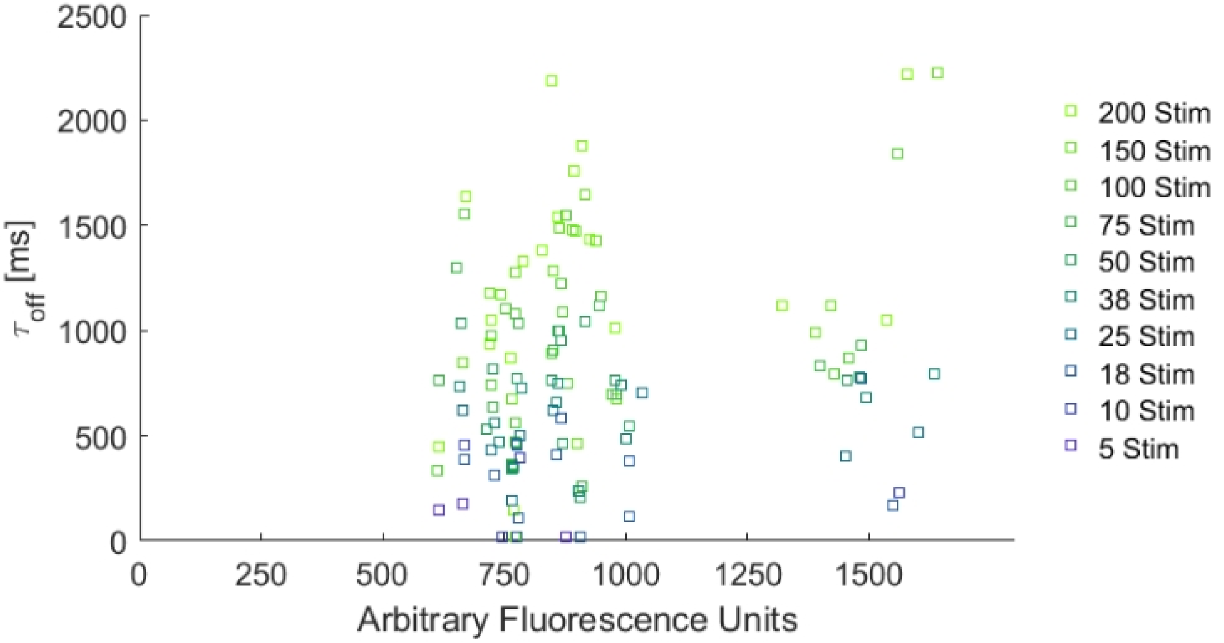

b

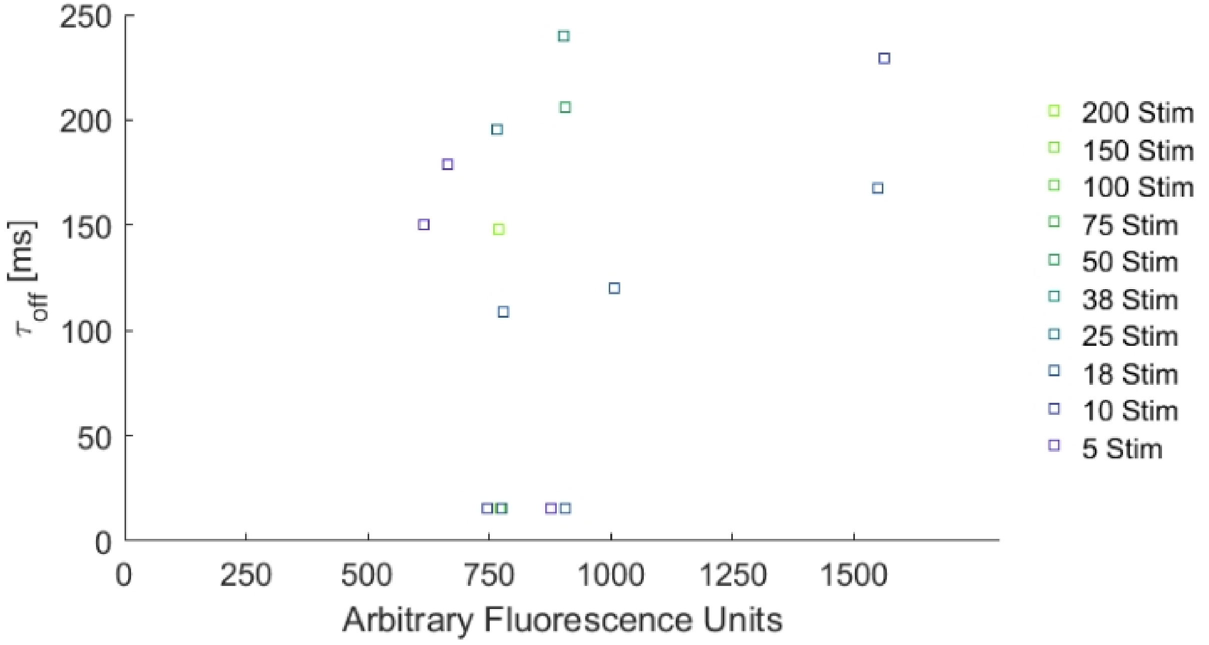

Supplementary Figure S3. Increasing prestimulus axon brightness is weakly associated with slowing of off time kinetics. Duration data from Fig. 3. (a,b) Each square represents the average off-time constant from each axon at a particular number of stimuli. (b) is the same data as (a), showing off time constants less than 250 ms. Time constants less than 125 ms should be interpreted with caution.

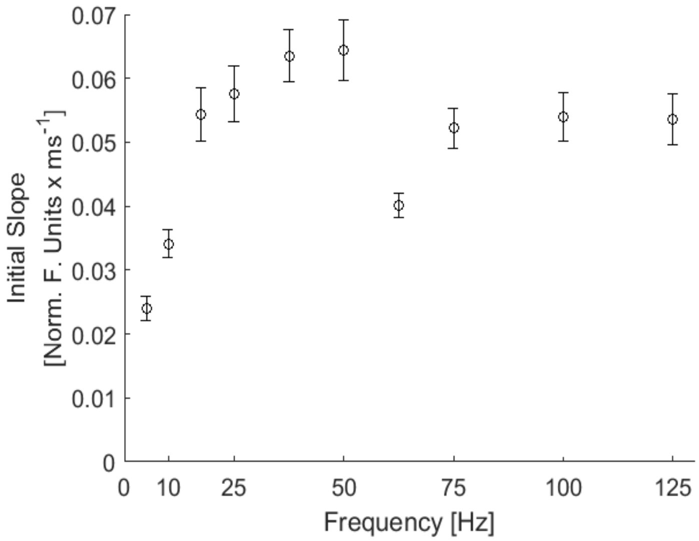

Supplementary Figure S4. Initial slope of the rise versus frequency suggests a small linear range for GCaMP6f in small fibers of the common peroneal nerve. (Norm. F. Units = Normalized Florescence Units). Frequency data from Fig. 4. (Kruskal-Wallis nonparametric analysis of variance, p=0.41).

Supplementary Video 1. GCaMP6f reports calcium transients in small diameter fibers of the peripheral nerve following electrical stimulation. Stimulus train is 100Hz for 1000ms.

Supplementary Table S1. Number of axons responsive to electrical stimulation used from each nerve.

Mouse	Nerve	Number of Axons
1	Left Common Peroneal	0
	Right Common Peroneal	1
2	Left Common Peroneal	0
	Right Common Peroneal	8
3	Left Common Peroneal	2
	Right Common Peroneal	3
4	Left Common Peroneal	1
	Right Common Peroneal	5
5	Left Common Peroneal	0
	Right Common Peroneal	3
Total		23

Supplementary Table S2. P-Values for multiple comparisons following Kruskal-Wallis test of maximum fluorescence change following small numbers of stimuli (data presented in Fig. 2c).

	3 Stim	4 Stim	5 Stim
2 Stim	0.00479	0.000178	1.08E-06
3 Stim		0.938	0.581
4 Stim			0.897

Supplementary Table S3. P-values for multiple comparisons following Kruskal-Wallis tests of calcium transients to stimulus trains of varying numbers of stimuli at 100 Hz. (a) P-values for multiple comparisons for maximum fluorescence change to pulses of varying numbers of stimuli (data presented in Fig. 3b). (b) P-values for multiple comparisons for on-time constants produced by single exponential fit of fluorescence change following a varying number of stimuli (data presented in Fig. 3d). (c) P-values for multiple comparisons for off-time constants produced by single exponential fit of fluorescence change following a varying number of stimuli (data presented in Fig. 3e).

(a)

	5 Stim	10 Stim	18 Stim	25 Stim	38 Stim	50 Stim	75 Stim	100 Stim	150 Stim	200 Stim
1 Stim	0.967	0.265	0.0891	0.0224	0.0023	5.30E-4	7.29E-4	3.53E-5	1.21E-5	4.56E-6
5 Stim		0.565	0.116	0.00827	7.83E-5	1.19E-6	5.99E-6	1.73E-7	1.72E-7	1.72E-7
10 Stim			0.999	0.855	0.149	0.0154	0.0333	1.15E-4	1.40E-5	1.99E-6
18 Stim				0.999	0.623	0.177	0.265	0.00388	6.27E-4	1.05E-4
25 Stim					0.979	0.714	0.796	0.0644	0.015	0.00336
38 Stim						1.00	1.00	0.798	0.489	0.239
50 Stim							1.00	0.96	0.749	0.439
75 Stim								0.981	0.85	0.595
100 Stim									1.00	0.997
150 Stim										1.00

(b)

	10 Stim	18 Stim	25 Stim	38 Stim	50 Stim	75 Stim	100 Stim	150 Stim	200 Stim
5 Stim	0.999	0.980	1.00	1.00	1.00	0.999	0.701	0.414	0.564
10 Stim			1.00	0.993	0.797	0.619	0.0434	0.00885	0.0216
18 Stim			1.00	0.761	0.0967	0.0487	8.99E-06	2.54E-07	1.85E-06
25 Stim				0.993	0.591	0.372	0.00135	5.79E-05	0.000376
38 Stim					0.989	0.904	0.0203	0.00109	0.00629
50 Stim						1.00	0.167	0.013	0.0626
75 Stim							0.713	0.227	0.473
100 Stim								0.998	1.00
150 Stim									1.00

(c)

	10 Stim	18 Stim	25 Stim	38 Stim	50 Stim	75 Stim	100 Stim	150 Stim	200 Stim
5 Stim	1.00	1.00	0.983	0.867	0.475	0.312	0.0401	0.0112	0.00336
10 Stim		1.00	0.999	0.959	0.624	0.429	0.0498	0.0121	0.00313
18 Stim			0.998	0.871	0.195	9.78E-02	0.000368	1.56E-05	1.08E-06
25 Stim				1.00	0.925	7.56E-01	0.085	0.0145	0.00256
38 Stim					0.994	0.926	0.131	0.0182	0.00247
50 Stim						1.00	0.542	0.128	0.0218
75 Stim							0.96	0.648	0.281
100 Stim								0.999	0.936
150 Stim									1.00

Supplementary Table S4. Correlation between pre-stimulus axon brightness and decay constant following constant frequency stimulus of various numbers of stimuli. Spearman's rank correlation coefficient (ρ) for each group of stimuli presented in Supplementary Figure S3. Significance assessed using the Benjamini-Hochberg method for controlling the false detection rate under multiple comparisons. No coefficient was determined to be significant.

Number of Stimuli	Spearman's ρ	p-value
5	-0.333	1.000
10	-0.333	0.750
18	0.0458	0.891
25	0.0714	0.905
38	0.205	0.367
50	-0.0737	0.677
75	0.0513	0.858
100	0.126	0.461
150	0.242	0.146
200	0.357	0.0344

Supplementary Table S5. P-values for multiple comparisons following Kruskal-Wallis tests of calcium transients to 2s stimulus trains of varying frequency. (a) P-values for multiple comparisons for on-time constants produced by single exponential fit of fluorescence change following stimulus (data presented in Fig. 4c). (b) P-values for multiple comparisons for off-time constants produced by single exponential fit of fluorescence change following stimulus (data presented in Fig. 4d).

(a)

	10 Hz	17.5 Hz	25 Hz	37.5 Hz	50 Hz	62.5 Hz	75 Hz	100 Hz	125 Hz
5 Hz	1.00	1.00	1.00	0.955	0.438	0.227	0.544	0.330	0.338
10 Hz		1.00	0.942	0.383	0.0273	0.00653	0.0508	0.0144	0.0166
17.5 Hz			0.973	0.461	0.0341	0.00792	0.0641	0.0178	0.0207
25 Hz				0.995	0.580	0.302	0.704	0.443	0.456
37.5 Hz					0.979	0.856	0.993	0.941	0.941
50 Hz						1.00	1.00	1.00	1.00
62.5 Hz							1.00	1.00	1.00
75 Hz								1.00	1.00
100 Hz									1.00

(b)

	10 Hz	17.5 Hz	25 Hz	37.5 Hz	50 Hz	62.5 Hz	75 Hz	100 Hz	125 Hz
5 Hz	1.00	0.937	0.117	0.216	0.421	0.310	0.0417	0.554	0.623
10 Hz		0.735	0.0124	0.0376	0.105	0.0657	0.00238	0.208	0.278
17.5 Hz			0.811	0.928	0.995	0.976	0.537	0.999	1.00
25 Hz				1.00	0.998	1.00	1.00	0.997	0.996
37.5 Hz					1.00	1.00	1.00	1.00	1.00
50 Hz						1.00	0.965	1.00	1.00
62.5 Hz							0.996	1.00	1.00
75 Hz								0.958	0.950
100 Hz									1.00
